# Characterization and Genomic Analysis of SFPH2, a Novel *T7virus* Infecting *Shigella*

**DOI:** 10.3389/fmicb.2018.03027

**Published:** 2018-12-14

**Authors:** Chaojie Yang, Haiying Wang, Hui Ma, Renlong Bao, Hongbo Liu, Lang Yang, Beibei Liang, Leili Jia, Jing Xie, Ying Xiang, Nian Dong, Shaofu Qiu, Hongbin Song

**Affiliations:** ^1^Institute of Disease Control and Prevention of PLA, Beijing, China; ^2^Joint Service Academy, National Defence University of People’s Liberation Army, Beijing, China; ^3^The 6th Medical Center of Chinese PLA General Hospital, Beijing, China

**Keywords:** bacteriophage, *Shigella flexneri*, *T7virus*, genome analysis, tail fiber

## Abstract

Shigellosis, caused by *Shigella*, is a major global health concern, with nearly 164.7 million cases and over a million deaths occurring annually worldwide. *Shigella flexneri* is one of the most common subgroups of *Shigella* with a high incidence of multidrug-resistance. The phage therapy approach is an effective method for controlling multidrug-resistant bacteria. However, only a few *Shigella* phages have been described to date. In this study, a novel lytic bacteriophage SFPH2 was isolated from a sewage sample obtained from a hospital in Beijing, China, using a multidrug-resistant *S. flexneri* 2a strain (SF2) isolated from the fecal sample of a dysentery patient. SFPH2 is a member of the *Podoviridae* virus family with an icosahedral capsid and a short, non-contractile tail. It was found to be stable over a wide range of temperatures (4–50°C) and pH values (pH 3–11). Moreover, SFPH2 could infect two other *S. flexneri* serotypes (serotypes 2 variant and Y). High-throughput sequencing revealed that SFPH2 has a linear double-stranded DNA genome of 40,387 bp with 50 open reading frames. No tRNA genes were identified in the genome. Comparative analysis of the genome revealed that the SFPH2 belongs to the subfamily *Autographivirinae* and genus *T7virus*. The genome shows high similarity with other enterobacterial *T7virus* bacteriophages such as *Citrobacter* phage SH4 (95% identity and 89% coverage) and *Cronobacter* phage Dev2 (94% identity and 92% coverage). A comparison of the fiber proteins showed that minor differences in the amino acid residues might specify different protein binding regions and determine host species. In conclusion, this is the first report of a *T7virus* that can infect *Shigella*; SFPH2 has a functional stability under a wide range of temperatures and pH values, showing the potential to be widely applied to control *Shigella*–associated clinical infections and reduce the transmission rates of *S. flexneri* serotype 2a and its variants in the environment.

## Introduction

Shigellosis, an infectious diarrheal disease, is caused by the enteric pathogen *Shigella*. It is a major worldwide health burden and causes nearly 164.7 million cases and over a million deaths every year, most of them occurring in developing countries (Kotloff et al., 1999). *Shigella flexneri*, one of the four *Shigella* subgroups, is the most common cause of endemic shigellosis (Kotloff et al., 1999; von Seidlein et al., 2006). Based on its serology, *S. flexneri* can be further subdivided into at least 20 serotypes (serotypes 1a, 1b, 1c, 1d, 2a, 2b, 2 variant, 3a, 3b, 4a, 4av, 4b, 5a, 5b, X, Xv, Y, Yv, F6, and 7b) (Qiu et al., 2013; Sun et al., 2013). Serotypes 2 variant, Xv, and Yv are newly reported serotypes and are associated with epidemic-level disease (Ye et al., 2010; Sun et al., 2013; Qiu et al., 2015). Serotype 2 of *S. flexneri* (2a, 2b, and 2 variant) is the most prevalent among the 20 serotypes (Qiu et al., 2015) and has been recently reported to show a high frequency of multidrug-resistance (Zhang et al., 2011; Cui et al., 2015), which undoubtedly narrows the choice of effective antimicrobials. In February 2017, the World Health Organization designated *Shigella* as the priority target for the development of new antimicrobials (WHO, 2017). Therefore, the development of novel treatments or strategies has become an absolute necessity to control *Shigella* infections.

Phages are viruses that infect bacteria and are estimated to be the most abundant organisms on earth (Chibani-Chennoufi et al., 2004). Phage therapy has proven to be an effective method to control bacterial infection (Sulakvelidze et al., 2001) and it has been successfully applied for the treatment of *Shigella dysenteriae* infections in children (d’Herelle, 1931). However, relatively few studies aimed at isolating or characterizing *Shigella* phages have been performed. Only ∼42 genome sequences of *Shigella* phages have been deposited with the NCBI nucleotide database and detailed studies of these phages are limited. In contrast, over 1,000 *Escherichia* and *Salmonella* phage sequences are readily available in the database. Phage host range is a significant feature of phage therapy and generally indicates the types (strains or species) of bacteria that the phage is able to infect (Hyman and Abedon, 2010). The tail fiber proteins of phages are thought to determine their host range and play an important role during the host recognition process (Garcia-Doval and van Raaij, 2013).

In this study, we report a novel *Shigella* lytic bacteriophage, SFPH2, which was isolated from a sewage sample using a multidrug-resistant *S. flexneri* 2a strain. We examined its biological properties, determined its genomic sequence, and compared its genome and tail fiber protein sequences with other known phage sequences. To the best of our knowledge, this is the first report of a *T7virus* able to infect *Shigella.* Our research will contribute to the understanding of *Shigella* phages and the effective control of Shigellosis caused by *Shigella*.

## Materials and Methods

### Bacterial Strains and Serotyping

The *S. flexneri* strains used in this study were isolated from the fecal samples of patients with diarrhea or dysentery. The fecal samples were screened for *Shigella* species as follows: samples were streaked onto *Salmonella*–*Shigella* (SS) agar and then incubated overnight at 37°C. Suspected *Shigella* colonies were picked and streaked directly onto SS agar and incubated overnight at 37°C. The resulting colonies were sub-cultured on Luria–Bertani (LB) agar plates and grown in a 37°C incubator. API 20E test strips (bioMérieux Vitek, Marcy-L’Etoile, France) were used for the identification of the *Shigella* strains following the manufacturer’s recommendations. The specific serotypes of the strains were identified using monovalent antisera (Denka Seiken, Tokyo, Japan).

Informed and written consents were obtained from the patients for sample collection and usage, as well as for the publication of obtained results in this study. All experiments were approved and authorized by the Ethics Committees of the Institute of Disease Prevention and Control, People’s Liberation Army, China.

### Phage Isolation and Purification

Bacteriophage SFPH2 was isolated from a sewage sample obtained from the 307 Hospital of PLA in Beijing, China. The bacteriophage was purified, using the agar double-layer method (Kropinski et al., 2009), from a multidrug-resistant *S. flexneri* 2a strain (SF2) isolated from a fecal sample of a 20-month-old male child diagnosed with diarrhea.

Sodium chloride (1 mol/L) and polyethylene glycol (PEG) 8000 (at a final concentration of 10%) were added to the sewage samples and incubated at 4°C for 24 h. Following centrifugation (8,000 ×*g* for 10 min at 4°C), the supernatant was filtered using a 0.22-μm membrane filter. Phages were pelleted by centrifugation at 12,000 ×*g* for 1 h at 4°C and resuspended in sterilized sodium chloride-magnesium sulfate (SM) buffer (100 mmol/L NaCl, 8 mmol/L MgSO_4_, 2% gelatin, and 50 mmol/L Tris–HCl [pH 7.5]). Spot tests were performed by spotting 0.05 mL of the phage solution onto bacterial lawns on a Luria–Bertani agar plate. Clear zone formations were assessed after overnight incubation.

To purify the phages, the double-layer agar plate method was used to collect single-plaque isolations, which were harvested and suspended in 0.5 mL of SM buffer. These steps were carried out at least five times in order to purify the phage. To concentrate the phage solution, 100 μL of the purified phage solution was added to 5 mL of host bacterial culture fluid (LB medium, OD_600_ = 0.5) and incubated at 37°C for 12 h. Culture samples were centrifuged at 12,000 ×*g* at 4°C for 10 min and filtered to remove cell debris. PEG 8000 (at a final concentration of 10%) was added to the phage solution and the mixture was centrifuged at 12,000 ×*g* at 4°C for 10 min. Subsequently, the solution was incubated overnight at 4°C. The samples were then resuspended in 0.5 mL of SM buffer and the concentrated phage solution was stored at 4°C.

### Phage Host Range Test

A host range test was performed with SFPH2 on 131 *S. flexneri*, 20 *Escherichia coli*, 12 *Salmonella typhimurium*, one *Salmonella enteritidis*, and three *Citrobacter freundii* strains isolated from the fecal samples of patients with diarrhea or dysentery. The sensitivity of the clinical strains to the phage was tested by the double-layer agar plate method, which was repeated three times. All the bacterial strains to be tested were grown overnight (14 h) at 37°C and 200 μl of each of those cultures was used in double layer plaque assays together with 100 μl of diluted phage lysate. The phage lysates were diluted from the phage stock and the titer ranged from 10^2^ to 10^4^ PFU/ml. In order to investigate the ability of SFPH2 to produce progeny in different strains, the efficiency of plating (EOP = phage titer on target bacteria/phage titer on host bacteria) was calculated as previously described (Khan Mirzaei and Nilsson, 2015).

### Transmission Electron Microscopy

Purified phages (approx. 10^10^ PFU/mL) were applied to a carbon-coated copper grid and stained with 2% (w/v) uranyl acetate for 40 s. Prepared phage samples were viewed using a transmission electron microscope (JEOL JEM-1200EX, Tokyo, Japan) at 100 kV.

### Thermal and pH Stability Tests

For thermal stability assessment, the phage preparation (1 × 10^10^ PFU/mL) was incubated at 37, 50, or 70°C for 24 h. For pH stability studies, phage at 1 × 10^10^ PFU/mL was incubated at pH 2–13 for 24 h. For all experiments, samples were taken at the 1, 3, 6, 9, and 24 h time-points and the phage titer was determined using the double-agar-layer method.

### One-Step Growth Curve

To determine the optimal MOI, serial dilutions of *S. flexneri* strain SF2 in exponential growth phase were added to aliquots of the SFPH2 stock solution so that the ratio of bacteriophage to host strain was 100, 10, 1, 0.1, and 0.01, respectively. After 5 min of adsorption, free bacteriophages were removed by centrifugation at 12,000 × g for 30 s, pellets were resuspended in LB medium, and samples were analyzed for bacteriophage titer using the double-layer agar technique after seven hours of incubation at 37°C. The solution with a ratio of bacteriophage to host strain that had the highest bacteriophage titer was chosen as having the optimal MOI. To obtain the one-step growth curve, the phage was mixed with the host strain in the exponential phase at the optimal MOI and incubated at 37°C for 5 min, then centrifuged at 12,000 ×*g* for 30 s to remove unabsorbed free phage. The precipitate was washed with LB broth (37°C) and then transferred into 20 mL of LB broth followed by incubation at 37°C. This time-point was defined as *t* = 0 s and every 10 min thereafter, a 0.5 mL sample was collected for a total duration of 100 min. The titration of phage particles was conducted using the double-layer agar method. The experiment was repeated three times.

### Phage DNA Preparation and Sequencing Analysis

The genomic DNA of phage SFPH2 was extracted following the method described by Wilcox et al. (Wilcox et al., 1996); the genome was sequenced using an Illumina Genome analyzer (Illumina, Inc., San Diego, CA, United States) with approximately 245-fold coverage. Gap filling was carried out using standard PCR and subsequent Sanger sequencing. The whole-genome sequence was analyzed using the CLC Genomics Workbench 9.0.1 (CLC Bio, Qiagen Bioinformatics, Germany). ORFs were predicted using the NCBI ORF Finder (NCBI Resource Coordinators, 2018) and the complete phage sequence was annotated using the online annotation server RAST (Aziz et al., 2008). The tRNAscan-SE software was used to search for putative tRNA genes (Lowe and Eddy, 1997). A map of the complete SFPH2 genome was constructed using SnapGene software 3.2.1 (GSL Biotech, United States). Sequence comparisons and sequence map generation were performed using BLAST^1^ and Easyfig (Sullivan et al., 2011), respectively. Sequence comparison of tail fiber proteins was performed using the online PredictProtein Tool (Yachdav et al., 2014).

### Nucleotide Sequence Accession Number

The complete genome sequence of SFPH2 is accessible at the GenBank database under accession number MH464253.

## Results

### Bacterial Strains

A total of 131 *S. flexneri* strains belonging to 15 serotypes were isolated from the fecal samples of patients with diarrhea or dysentery (Table 1). Of these, one serotype 2a strain, SF2, was identified as a multidrug-resistant bacterium, with resistance to aminoglycosides, amphenicols, penicillins, quinolones, and tetracyclines. This SF2 strain was used to isolate the bacteriophage, SFPH2. The remaining 130 strains were used to determine the host range.

**Table 1:** Host range of *Shigella* phage SFPH2.

*S. fexneri* serotypes	Spectrum^∗^	EOP	Notes†
1a	0/6	–	–
1b	0/5	–	–
1c	0/10	–	–
2a	25/25	0.36–1	Medium/High production
2b	0/6	–	–
2 variant	8/10	0.14–0.25	Medium production
3a	0/6	–	–
3b	0/4	–	–
4a	0/10	–	–
4av	0/15	–	–
4b	0/6	–	–
X	0/10	–	–
Xv	0/6	–	–
Y	3/8	0.16–0.23	Medium production
F6	0/4	–	–


*“^∗^” The numerator represents the number of strains that can be infected by SFPH2. The denominator represents the total number of strains used in this test. “†” EOP > 0.5: High production; 0.1 < EOP < 0.5: Medium production (Viazis et al., 2011). The EOP values on each strain are listed in Supplementary Table S1.*

### Morphology and Host Range Test

**FIGURE 1:**
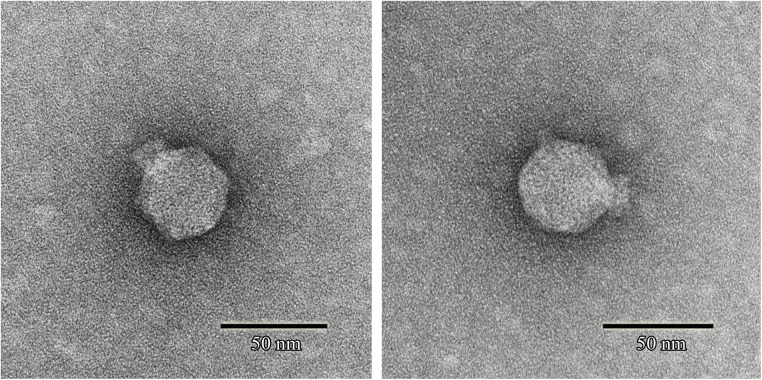
Transmission electron micrograph of *Shigella* phage SFPH2. Phage SFPH2 was negatively stained with 2% (w/v) uranyl acetate solution for 40 s on a copper grid. It has an icosahedral capsid, ∼50 nm in diameter, and a short non-contractile tail. Bar corresponds to 50 nm.

Phage SFPH2 formed clear plaques, ∼2 mm in diameter, on its host bacterial strain on a double-layered agar plate following 6-h incubation at 37°C. Transmission electron microscopy of the purified and concentrated phage particles showed that phage SFPH2 has an icosahedral capsid, ∼50 nm in diameter, and a short non-contractile tail (Figure 1), matching the typical morphological features of the *Podoviridae* virus family.

A total of 131 *S. flexneri* strains belonging to 15 serotypes were used to determine the host range of SFPH2. All 25 *S. flexneri* serotype 2a strains and a number of serotype 2 variant and Y strains could be lysed by SFPH2, suggesting that this phage has a broad host range in *S. flexneri* 2a strains and limited host range in serotype 2 variant and Y strains (Table 1). Results of the EOP value of SFPH2 on these strains indicated that the phage could cause “medium / high production” efficiency on *S. flexneri* serotype 2a strains and “medium production” efficiency on serotype 2 variant and Y strains according to the classification rules of EOP (Viazis et al., 2011; Table 1 and Supplementary Table S1). In order to test whether SFPH2 can infect other species of bacteria, 20 *Escherichia coli*, 12 *Salmonella typhimurium*, one *Salmonella enteritidis*, and three *Citrobacter freundii* strains were also used to perform the host range test. However, SFPH2 could not lyse any of these strains (data not shown).

### Host Cell Lytic Activity Test

**FIGURE 2:**
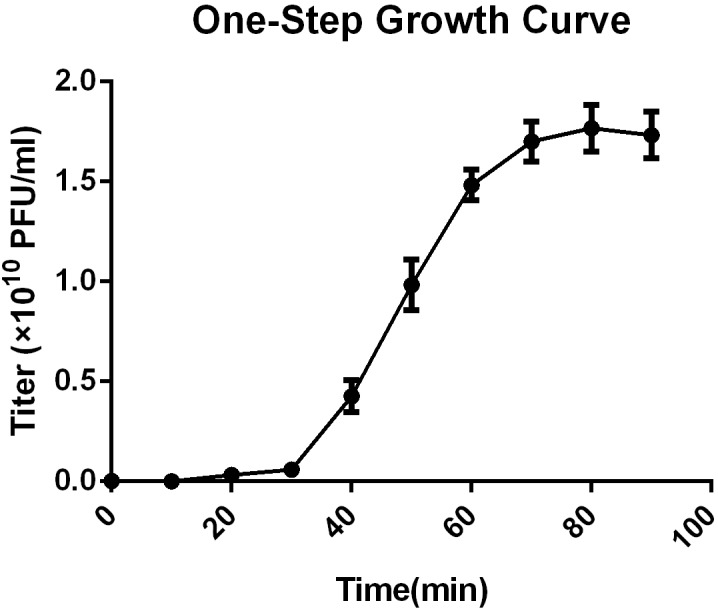
One-step growth curve of *Shigella* phage SFPH2. *S. flexneri* 2a strain SF2 was used as the host. All the samples were taken per 10 min and the phage titer was determined using the double-agar-layer method. The error bars indicate standard deviations.

The multiplicity of infection (MOI) test showed that an MOI of 0.1 yielded the highest level of phage progeny production, 1.62 × 10^10^ plaque forming units (PFU)/mL (Supplementary Table S2). This optimal MOI of SFPH2 was used to generate a one-step growth curve, which was conducted with its host strain SF2 and showed that SFPH2 has a 30-min latent period and a 40-min outbreak period (Figure 2). The maximum number of progeny released from one host bacterium was calculated as the phage burst titer (1.86 × 10^10^ PFU/mL) divided by the bacterial infection titer (6.23 × 10^8^ colony forming units (CFU)/mL) and was estimated to be 30 PFU/cell.

### Thermal and pH Stability Test

**FIGURE 3:**
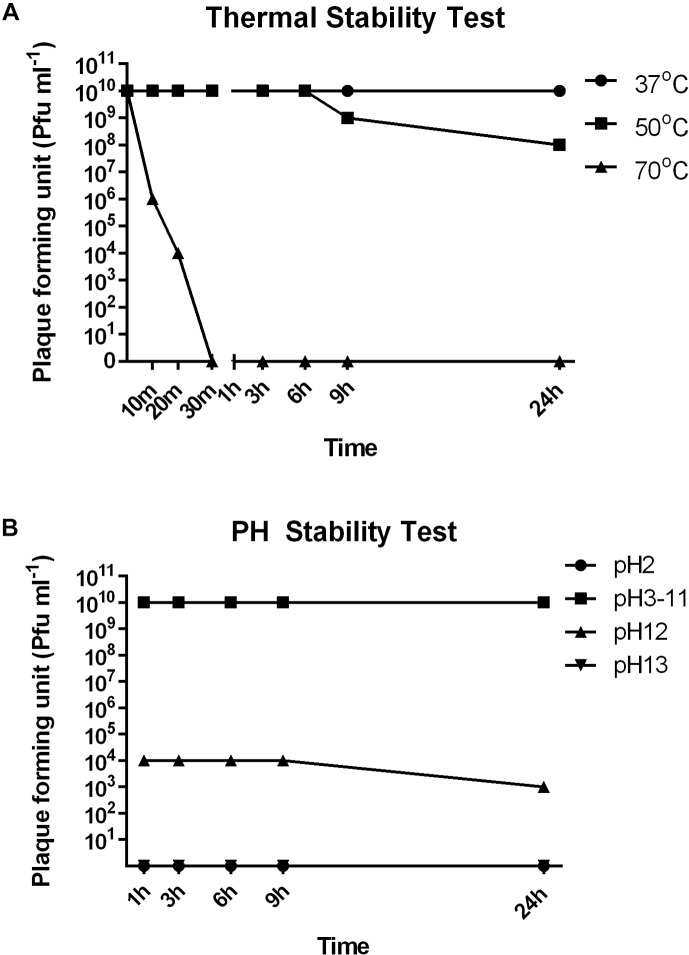
Thermal and pH stability of *Shigella* phage SFPH2. *S. flexneri* 2a strain SF2 was used as the host. **(A)** Thermal stability test. Phage SFPH2 preparation (1 × 10^10^ Pfu/ml) was incubated at 37, 50, or 70°C for 24 h. **(B)** PH stability test. Phage SFPH2 at 1 × 10^10^ Pfu/ml was incubated at pH 2–13 for 24 h. All the samples of these two tests were taken at 1, 3, 6, 9, and 24 h time-points and the phage titer was determined using the double-agar-layer method.

To investigate the stability of SFPH2 under various environmental conditions, we analyzed its stability at different temperatures and pH values. SFPH2 retained high activity following incubation at 37°C and nearly 80% of the phages were viable after 24 h incubation at 50°C (Figure 3A). However, no active phages were found after 30 min at 70°C. In the pH stability test, SFPH2 phage remained unaffected over a pH range of 3–11 after 24 h, exhibiting high stability within this pH range. At pH 12, 30–40% of the phages were still viable. However, it showed 0 titer at pH 2 and 13 (Figure 3B).

### Characterization of the Phage SFPH2 Genome

**FIGURE 4:**
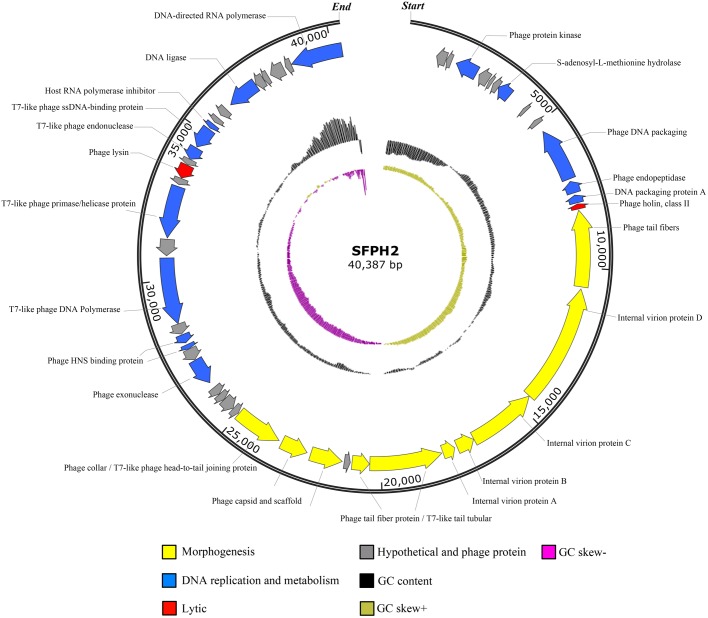
Genome map of the *Shigella* phage SFPH2 genome. A genome map of the *S. flexneri* phage SFPH2 illustrates putative open reading frames (ORFs) along with the direction of transcription represented by arrows. Functional proteins, GC content and GC skew are depicted by different colors.

Phage SFPH2 has a linear double-stranded DNA genome, 40,387 bp in length with a G+C composition of 52.4%. Of the 50 total putative open reading frames (ORFs), nine (18%) denote hypothetical proteins and 14 (28%) were designated as phage proteins with no specific functional annotations. Twenty-seven ORFs likely encode functional proteins. These ORFs were divided into three putative functional protein groups: (i) lytic-related proteins (such as gp38, Phage lysin, N-acetylmuramoyl-L-alanine amidase), (ii) DNA replication and metabolism-related proteins (such as gp29, phage exonuclease; gp34, T7-like phage DNA polymerase; gp36, T7-like phage primase/helicase protein; gp40, T7-like phage endonuclease; gp45, DNA ligase), and (iii) morphogenesis-related proteins (such as gp14, phage tail fibers; gp19 and gp20, T7-like tail tubular; gp22 and gp23, phage capsid and scaffold; gp24, phage collar/T7-like phage head-to-tail joining protein) (Figure 4). No tRNA genes were found in the genome.

**FIGURE 5:**
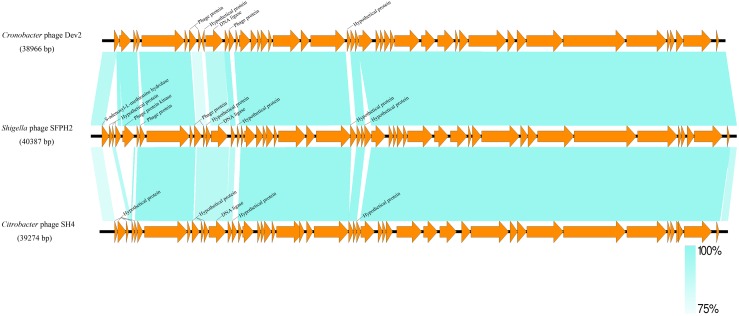
The genome comparison of *Shigella* phage SFPH2, *Citrobacter* phage SH4 and *Cronobacter* phage Dev2. Green shading denotes regions of shared homology amongst the different genomes. Only the unique ORFs and the ORFs with less than 90% homology were marked.

A similarity comparison with phage proteins in the NCBI database showed that the SFPH2 genome belongs to the subfamily *Autographivirinae* in the family *Podoviridae*, with the highest similarity to the T7 branch. The highest similarity throughout was observed with the enterobacterial *T7virus*, *Citrobacter* phage SH4 (95% identity and 89% coverage, GenBank accession no. KU687350) and *Cronobacter* phage Dev2 (94% identity and 92% coverage, GenBank accession no. HG813241). The genome size (approx. 39–40 kb) of SFPH2 is very similar to that of the two *T7virus* genomes. SFPH2 was shown to have a linear genome by restriction enzyme digestion (data not shown), and the other two phages also have a linear genome. The genome comparison of these three phages showed that one encoded functional protein, viz. S-adenosyl-L-methionine hydrolase, and several hypothetical phage proteins are unique to SFPH2. The remaining ORFs are homologous or have high similarity to the *Citrobacter* phage SH4 and *Cronobacter* phage Dev2 genomes (Figure 5).

**FIGURE 6:**
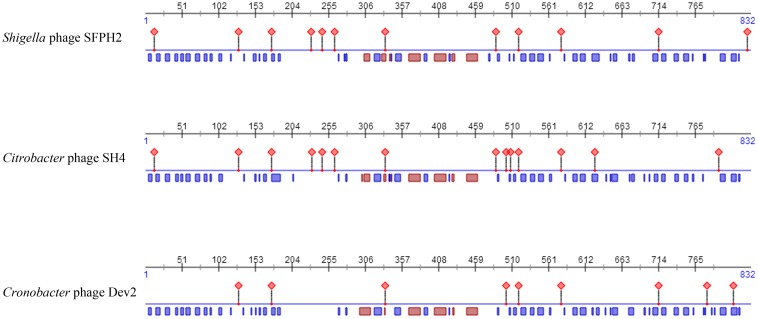
The predicted features comparison of fiber proteins of *Shigella* phage SFPH2, *Citrobacter* phage SH4 and *Cronobacter* phage Dev2. The red diamond represents protein-binding region; the red and blue rectangle represents the helix and strand of protein, respectively.

Moreover, as tail fiber proteins have been associated with host range determination, the sequences and predicted features of the tail fiber proteins of these three phages were also compared. The results showed that the tail fiber proteins have high similarities (SFPH2: SH4 97% similarity; SFPH2: Dev2 96% similarity) and that the position of the helix and strand in the protein are also substantially similar. However, 27 and 33 amino acid residues of the SFPH2 tail fiber protein sequence differ to those in phages SH4 and Dev2, which might result in differences in their protein binding regions (Figure 6 and Supplementary Figure S1).

## Discussion

Antimicrobial resistance (AMR) has been recognized as a significant global threat to human health (WHO, 2018). Moreover, the rate of multidrug resistance (MDR, resistance to three or more classes of antimicrobials) in *Enterobacteriaceae*, such as *Shigella*, is increasing alarmingly because of the widespread and indiscriminate use of antibiotics (Zhang et al., 2011; Nuesch-Inderbinen et al., 2016). In this study, we collected and analyzed 131 *S. flexneri* strains, 95% of which were found to be multidrug-resistant (data not shown). Phages have been proposed as alternative agents to protect against and treat infectious diseases caused by AMR pathogens (Sulakvelidze et al., 2001). The aim of this study was to isolate and characterize phage(s) that target multidrug-resistant *S. flexneri* and evaluate their effectiveness as antimicrobial agents to combat these bacteria.

**FIGURE 7:**
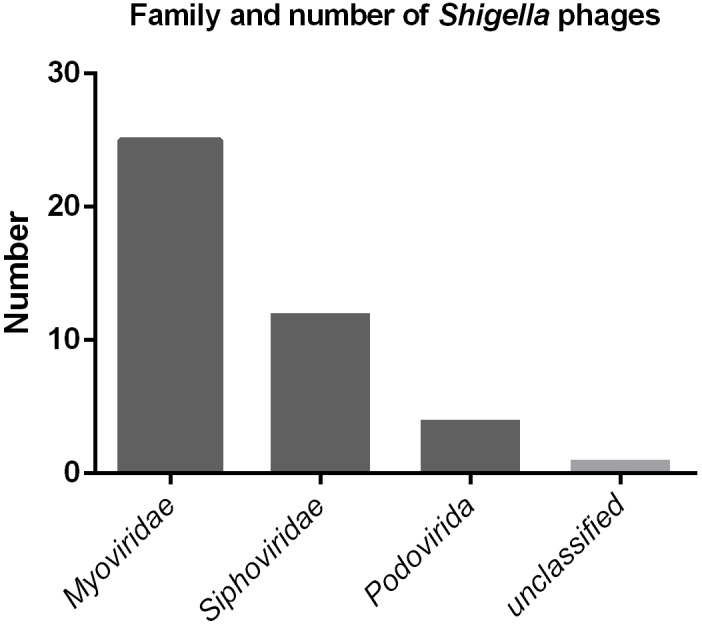
The family and number of *Shigella* phages in the NCBI database.

To date, a total of 42 *Shigella* phages are available in the NCBI database (Supplementary Table S3). The sizes of their genome range between 39.8 kbp and 170.7 kbp. According to the classification standards of the ICTV (International Committee on Taxonomy of Viruses) (ICTV, 2017), they all belong to the order *Caudovirales*. At the family level, there are 25, 12, and five *Shigella* phages belonging to the family *Myoviridae*, *Siphoviridae*, and *Podoviridae*, respectively (Figure 7). Of the five family *Podoviridae* phages, three belong to the subfamily *Sepvirinae* and one belongs to the subfamily *Enquartavirinae* (Gray et al., 2014; Jun et al., 2014). SFPH2 identified in this study is the only phage known to belong to the subfamily *Autographivirinae* and genus *T7virus* (Table 2). The genus *T7virus* was first accepted under the name *T7 phage group* and not assigned to any family according to the ICTV Second Report in 1976. The genus was classified into the family *Podoviridae* in 1981 and renamed twice as *T7-like phages* and *T7likevirus* by the ICTV in 1995 and 1999, respectively. The latest renaming of this genus, *T7virus*, was conducted in 2015. Although 42 years have passed since its first designation, only 88 complete *T7virus* genomes have been analyzed and submitted to the NCBI database (NCBI Resource Coordinators, 2018), including the genomes of *Citrobacter*, *Pseudomonas*, *Salmonella* and *Escherichia* phages. However, to date, no *Shigella* phage from this genus has been characterized. Given the large number of phages in nature and their powerful potential applications, further discovery and characterization of *Shigella* phages is essential.

**Table 2:** List of currently known *Shigella* phages of family *Podoviridae.*

Phage name	GenBank accession no.	Genome length (bp)	G+C content (%)	No. of CDSs	Genome type	Taxonomic Subfamily	Taxonomic Genera	Reference
*Shigella* phage pSb-1	KF620435	71629	42.7	103	circular	*Enquartavirinae*	*G7cvirus*	Jun et al., 2014
*Shigella* phage 75/02 Stx	KF766125	60875	49.1	76	circular	*Sepvirinae*	*Pocjvirus*	Unpublished
*Shigella* phage POCJ13	KJ603229	62699	49.3	79	linear	*Sepvirinae*	*Pocjvirus*	Gray et al., 2014
*Shigella* phage Ss-VASD	KR781488	62851	50.1	78	linear	*Sepvirinae*	*Tl2011virus*	Unpublished
*Shigella* phage SFPH2	MH464253	40387	52.4	50	linear	*Autographivirinae*	*T7virus*	This study


In the therapeutic application of bacteriophages, a broad host range is considered to be more advantageous, equivalent to broad-spectrum antibiotics (Ross et al., 2016). Moreover, the ability to continuously amplify phages themselves at the site of infection is a great advantage of phage therapy (Housby and Mann, 2009). But bacterial killing may induced by high multiplicity virion adsorption and that occurs without phage production, which is called “lysis from without” and happens when an overload of phages simultaneously infects a bacterium (Abedon, 2011). This is obviously less useful for phage therapy. Therefore, determining the infection and replication ability of phages to target strains is very important before their application for therapy. Efficiency of plating (EOP), which is frequently used to identify phages suitable for phage therapy, can reflect the ability of phages to produce progeny in bacteria (Khan Mirzaei and Nilsson, 2015). In this study, we found that SFPH2 was able to infect all serotype 2a and some serotype 2 variant and Y *S. flexneri* strains. The EOP value of SFPH2 showed it can keep multiplying within these strains. Therefore, this phage could potentially be used against infections caused by these three serotypes. Moreover, SFPH2 was unable to infect other bacteria, such as *E. coli*, ensuring that its therapeutic use would not affect non-pathogenic *E. coli* and likely other natural intestinal flora. A previous study has reported that *Shigella* transmission is particularly associated with poor sanitation because of the low infectious dose of this pathogen (Esrey et al., 1985). *Shigella flexneri* strains can survive for several months in contaminated water and foodstuffs (Islam et al., 1993, 1996). The *Shigella* phage could easily be released into the environment in areas where Shigellosis is endemic. Thus, a powerful tool, such as a bacteriophage, would be extremely useful for controlling the rapid spread of *Shigella*.

A detailed phage genome analysis provided molecular insight into the differences between SFPH2 and other enterobacterial *T7virus* phages. The SFPH2 genome has a similar size and high sequence similarity with *Citrobacter* phage SH4 and *Cronobacter* phage Dev2. Although the three phage genomes were found to be highly similar, their hosts belong to different species. It has been previously noted that host range is associated with the tail fiber proteins or receptor-binding proteins in some phages (Garcia-Doval and van Raaij, 2013). Sequence divergence in tail fiber proteins will lead to different host specificities. Phages can change or expand their host range by mutation of their tail fiber proteins (Casjens and Molineux, 2012). Our results show that the fiber protein sequences and polypeptide folds of the three *T7virus* phages are highly similar, except for 3–4% of the amino acid residues that differ; this may result in different protein binding regions and subsequently determine the range of host species. Seckler et al. also found that although the receptor-binding domains of three P22-like phages have no recognizable amino acid sequence similarity and the host ranges of the phages are different, all three have the same polypeptide fold (Barbirz et al., 2008; Muller et al., 2008). These results suggest that the interaction between the tail fiber protein and the host surface receptor occurs via more specific binding. However, most studies have only examined the conformation of tail fiber proteins and few studies have focused on progression of the binding mechanism between the tail fiber proteins and the receptor on the outer membrane surface of host bacteria. Clearly, mechanisms of host species determination and phage-receptor binding are far from being well understood and will remain fertile research ground for some time to come. Further research on these points will help to develop artificial broad host range phages against multiple bacteria.

## Conclusion

In conclusion, we have characterized and sequenced a novel *Shigella* phage, SFPH2, belonging to the family *Podoviridae*. We have shown that it lyses *S. flexneri* serotype 2a, 2 variant, and Y strains and is functionally stable under a wide range of temperatures and pH conditions. Therefore, it has the potential to be widely applied to control *Shigella*–associated clinical infections.

## Author Contributions

HS and SQ designed the experiments. HW, HM, RB, HL, and BL performed the experiments. LY, JX, and YX analyzed the data. ND and LJ prepared the tables and figures. CY and BL prepared the manuscript. All authors have read and approved the final manuscript.

## Conflict of Interest Statement

The authors declare that the research was conducted in the absence of any commercial or financial relationships that could be construed as a potential conflict of interest.

## Footnotes

1 http://blast.ncbi.nlm.nih.gov
