# Supplementary material for: Characterization and Genomic Analysis of SFPH2, a Novel T7virus Infecting Shigella
Source: Front Microbiol. 2018 Dec 14;9:3027. doi: 10.3389/fmicb.2018.03027 (PMC6302001; doi:10.3389/fmicb.2018.03027)
Supplement: Supplementary file 1 [file Data_Sheet_1.pdf]

Fig. S1 The comparison of fiber protein sequences of *Shigella* phage SFPH2, *Citrobacter* phage SH4 and *Cronobacter* phage Dev2

The "*" represents the same amino acid; The blank represents different amino acids.

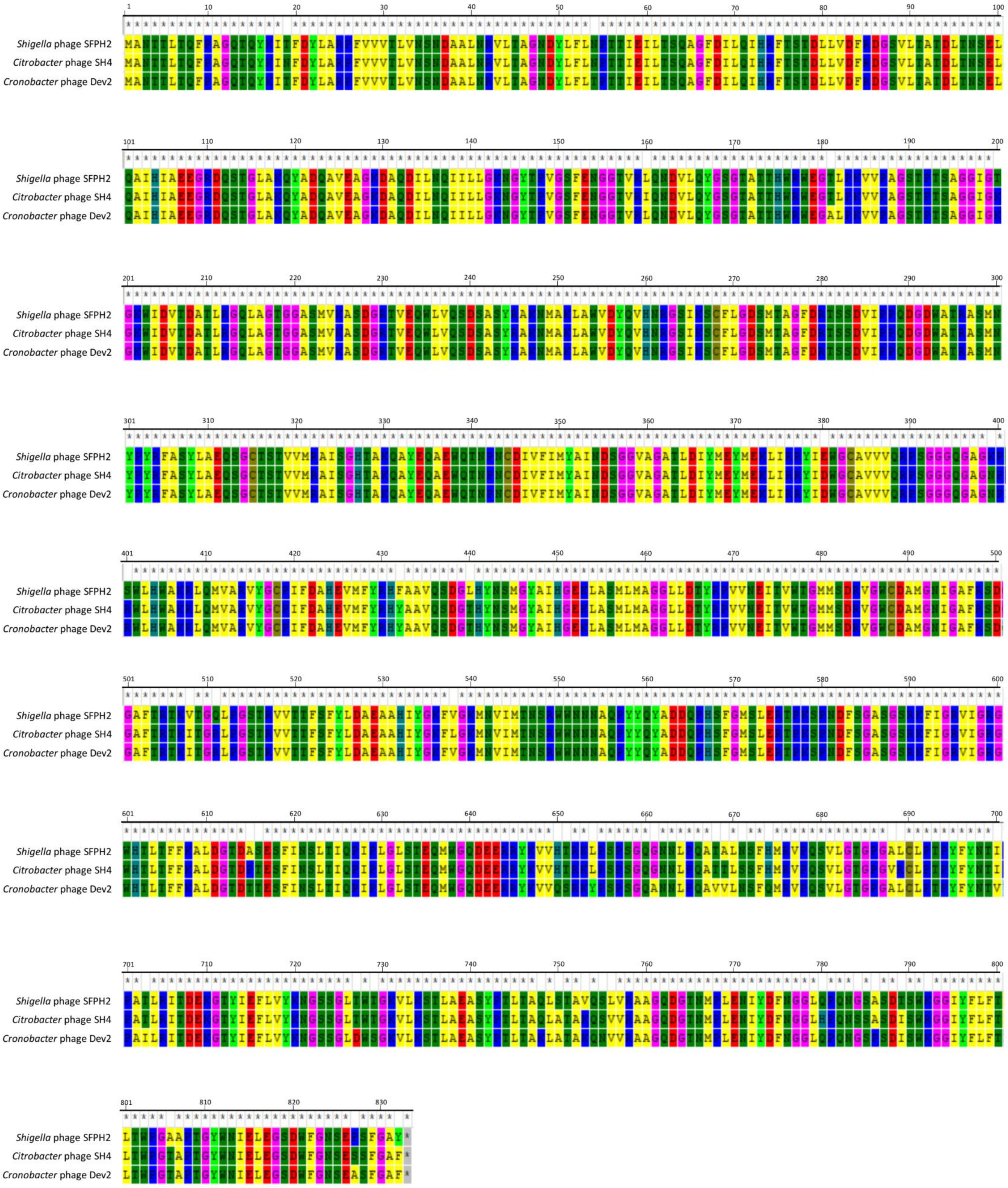

Table S1 The result of efficiency of plating (EOP) test

Strain No.	Serotypes	Efficiency of plating (EOP)	Notes
SF1	2a	0.83±0.03	High production
**SF2**	**2a**	**1**	High production
SF4	2a	0.54±0.06	High production
SF7	2a	0.43±0.11	Medium production
SF12	2a	0.47±0.05	Medium production
SF23	2a	0.58±0.13	High production
SH2a001	2a	0.60±0.12	High production
SH2a002	2a	0.67±0.06	High production
SH2a003	2a	0.43±0.17	Medium production
SH2a004	2a	0.39±0.15	Medium production
SH2a005	2a	0.55±0.03	High production
SH2a006	2a	0.67±0.16	High production
SH2a007	2a	0.72±0.12	High production
SH2a008	2a	0.62±0.09	High production
SH2a009	2a	0.41±0.21	Medium production
SH2a010	2a	0.59±0.09	High production
SH2a011	2a	0.36±0.05	Medium production
SH2a012	2a	0.88±0.06	High production
SH2a013	2a	0.61±0.03	High production
SH2a014	2a	0.56±0.14	High production
SH2a015	2a	0.42±0.04	Medium production
SH2a016	2a	0.74±0.07	High production
SH2a017	2a	0.37±0.04	Medium production
SH2a018	2a	0.61±0.07	High production
SF9	2 variant	0.15±0.04	Medium production
SF11	2 variant	0.14±0.11	Medium production
SF24	2 variant	0.25±0.07	Medium production
SH2c001	2 variant	0.16±0.09	Medium production
SH2c002	2 variant	0.18±0.12	Medium production
SH2c003	2 variant	0.24±0.06	Medium production
SH2c005	2 variant	0.20±0.03	Medium production
SH2c006	2 variant	0.17±0.09	Medium production
SHY002	Y	0.16±0.15	Medium production
SHY007	Y	0.18±0.08	Medium production
SHY008	Y	0.23±0.06	Medium production

EOP = phage titer on target bacteria / phage titer on host bacteria. EOP > 0.5: High production; 0.1 < EOP < 0.5: Medium production (Viazis et al., 2011). Each EOP value is the mean of three measurements, followed by its standard deviation. The plating on the host strain SF2 (EOP = 1.0) is marked in bold.

Table S2 The result of multiplicity of infection (MOI) test

Ratio*	Titer
100	6.23×10^{9}
10	5.89×10^{9}
1	7.21×10^{9}
0.1	1.62×10^{10}
0.01	1.04×10^{10}

The “*” represents the ratio of bacteriophage to host strain.

Table S3 List of *Shigella* phages on NCBI database

Phage name	GenBank accession no.	Genome length (bp)	Genome type	Taxonomy
Shigella phage SSP1	KY963424	113299 bp	linear	Viruses; dsDNA viruses, no RNA stage; Caudovirales; Siphoviridae.
Shigella phage SFN6B	KY684082	43036 bp	linear	Viruses; unclassified bacterial viruses
Shigella phage Sf20	MF327006	163982 bp	linear	Viruses; unclassified bacterial viruses
Shigella phage Sf21	MF327007	166002 bp	linear	Viruses; unclassified bacterial viruses
Shigella phage Sf25	MF327009	168573 bp	linear	Viruses; unclassified bacterial viruses.
Shigella phage Sf24	MF327008	168112 bp	linear	Viruses; unclassified bacterial viruses.
Shigella phage Sf17	MF327004	90092 bp	linear	Viruses; unclassified bacterial viruses.
Shigella phage Sf19	MF327005	90375 bp	linear	Viruses; dsDNA viruses, no RNA stage; Caudovirales; Myoviridae;Ounavirinae; Mooglevirus; unclassified Mooglevirus.
Shigella phage SHSML-45	KX130863	108050 bp	linear	Viruses; dsDNA viruses, no RNA stage; Caudovirales; Siphoviridae; T5virus; unclassified T5likevirus.
Shigella phage Sf14	MF327003	87575 bp	linear	Viruses; unclassified bacterial viruses.
Shigella phage vB_SflS-ISF001	MG049919	50552 bp	linear	Viruses; dsDNA viruses, no RNA stage; Caudovirales; Siphoviridae.
Shigella phage Sf23	MF158046	167678 bp	linear	Viruses; unclassified bacterial viruses.
Shigella phage Sf22	MF158045	166283 bp	linear	Viruses; unclassified bacterial viruses.
Shigella phage Sf18	MF158044	90270 bp	linear	Viruses; unclassified bacterial viruses.
Shigella phage Sf16	MF158043	88580 bp	linear	Viruses; unclassified bacterial viruses.
Shigella phage Sd1	MF158042	48262 bp	linear	Viruses; unclassified bacterial viruses.
Shigella phage Sf15	MF158041	88474 bp	linear	Viruses; unclassified bacterial viruses.
Shigella phage Sf13	MF158040	87570 bp	linear	Viruses; unclassified bacterial viruses.
Shigella phage Sf12	MF158039	47647 bp	linear	Viruses; unclassified bacterial viruses.
Shigella phage vB_SsoS-ISF002	MF093736	50564 bp	linear	Viruses; dsDNA viruses, no RNA stage; Caudovirales; Siphoviridae
Shigella phage Sf11	MF158038	46454 bp	linear	Viruses; unclassified bacterial viruses.
Shigella phage SH7	KX828711	164870 bp	linear	Viruses; dsDNA viruses, no RNA stage; Caudovirales; Myoviridae;Tevenvirinae; T4virus.
Shigella phage SH6	KX828710	50552 bp	linear	Viruses; dsDNA viruses, no RNA stage; Caudovirales; Siphoviridae;Tunavirinae; T1virus; unclassified T1virus.
Shigella phage SHSML-52-1	KX130865	169621 bp	linear	Viruses; dsDNA viruses, no RNA stage; Caudovirales; Myoviridae;Tevenvirinae; Rb69virus; unclassified Rb69virus.
Shigella phage SHBML-50-1	KX130864	166634 bp	linear	Viruses; dsDNA viruses, no RNA stage; Caudovirales; Myoviridae;Tevenvirinae; T4virus.
Shigella phage SHFML-26	KX130862	168993 bp	linear	Viruses; dsDNA viruses, no RNA stage; Caudovirales; Myoviridae;Tevenvirinae; T4virus.

Shigella phage SHFML-11	KX130861	170650 bp	linear	Viruses; dsDNA viruses, no RNA stage; Caudovirales; Myoviridae;Tevenvirinae; T4virus.
Shigella phage pSf-2	KP085586	50109 bp	circular	Viruses; dsDNA viruses, no RNA stage; Caudovirales; Siphoviridae;Tunavirinae; T1virus
Shigella phage pSs-1	KM501444	164999 bp	circular	Viruses; dsDNA viruses, no RNA stage; Caudovirales; Myoviridae;Tevenvirinae; T4virus.
Shigella phage Ss-VASD	KR781488	62851 bp	linear	Viruses; dsDNA viruses, no RNA stage; Caudovirales; Podoviridae;Sepvirinae; Tl2011virus
Shigella phage 75/02 Stx	KF766125	60875 bp	circular	Viruses; dsDNA viruses, no RNA stage; Caudovirales; Podoviridae;Sepvirinae; Pocjvirus.
Shigella phage Shf125875	KM407600	169062 bp	linear	Viruses; dsDNA viruses, no RNA stage; Caudovirales; Myoviridae;Tevenvirinae; Rb69virus
Shigella phage pSb-1	KF620435	71629 bp	circular	Viruses; dsDNA viruses, no RNA stage; Caudovirales; Podoviridae;G7cvirus.
Shigella phage POCJ13	KJ603229	62699 bp	linear	Viruses; dsDNA viruses, no RNA stage; Caudovirales; Podoviridae;Sepvirinae; Pocjvirus.
Shigella phage pSf-1	KC710998	51821 bp	linear	Viruses; dsDNA viruses, no RNA stage; Caudovirales; Siphoviridae
Shigella phage SfIV	KC814930	39758 bp	linear	Viruses; dsDNA viruses, no RNA stage; Caudovirales; Myoviridae
Shigella phage SfII	KC736978	41475 bp	linear	Viruses; dsDNA viruses, no RNA stage; Caudovirales; Myoviridae
Shigella phage EP23	JN984867	44077 bp	linear	Viruses; dsDNA viruses, no RNA stage; Caudovirales; Siphoviridae; Hk578virus
Shigella phage phiSboM-AG3	FJ373894	158006 bp	linear	Viruses; dsDNA viruses, no RNA stage; Caudovirales; Ackermannviridae; Aglimvirinae; Ag3virus.
Shigella phage Shfl2	HM035025	165919 bp	circular	Viruses; dsDNA viruses, no RNA stage; Caudovirales; Myoviridae; Tevenvirinae; T4virus
Shigella phage Shfl1	HM035024	50661 bp	circular	Viruses; dsDNA viruses, no RNA stage; Caudovirales; Siphoviridae; Tunavirinae; T1virus.
Shigella phage SP18	GQ981382	170605 bp	linear	Viruses; dsDNA viruses, no RNA stage; Caudovirales; Myoviridae; Tevenvirinae; Sp18virus
